# Numerical investigation on the mechanical response of steel-frame polyethylene pipelines subjected to strike-slip faulting

**DOI:** 10.1371/journal.pone.0353153

**Published:** 2026-07-29

**Authors:** ZhaoLiang Zhu, Xin Huang

**Affiliations:** College of Mechanical and Electrical Engineering, Yibin University, Yibin, China; Gdańsk University of Technology: Politechnika Gdanska, POLAND

## Abstract

This study investigates the failure behavior of buried steel-reinforced polyethylene (SRPE) pipelines crossing strike-slip faults. A high-fidelity three-dimensional pipe-soil interaction model is established using the finite element method, and layered modeling with tie constraints is adopted to replicate the synergistic mechanical characteristics between the PE matrix and steel frame. The effects of pipe-fault intersection angle, steel wire diameter, and soil type on the mechanical response, buckling morphology, and critical strain of the pipeline are systematically examined, and the applicability of three design codes (CSA Z662-2023, GB 50470−2017, EN 13476−3) is quantitatively evaluated. The results show that SRPE pipelines exhibit a three-stage mechanical behavior under fault displacement: elastic bending at small displacement, plastic buckling propagation at moderate displacement, and sectional distortion with global instability at large displacement. The steel frame and PE matrix form an efficient synergistic mechanism featured by “matrix energy dissipation and frame load-bearing”, where failure initiates from plastic deformation of the PE matrix and further induces steel frame yielding and pipeline leakage. The pipe-fault angle dominates the loading pattern: tension is dominant at 30°, while transverse compression at 150° represents the most hazardous condition. The optimal wire diameter ranges from 2.0 to2.5mm; loess provides the strongest constraint, whereas sand is the weakest. Conventional constant strain criteria neglect the angle effect and show obvious limitations in engineering practice. The findings provide significant theoretical support for the seismic design and safety assessment of SRPE pipelines crossing active fault zones.

## 1. Introduction

Buried pipelines constitute the principal transportation infrastructure for strategic resources like oil and natural gas, and are acclaimed as the “lifeline projects” for energy transmission. The secure operation of these pipelines is directly tied to the stability of national energy supplies and public safety. Long-distance pipelines inevitably traverse geologically active regions, where active faults represent the primary geological hazard capable of causing pipeline damage [1]. The horizontal displacement associated with strike-slip faults subjects pipelines to a combination of tensile, compressive, and bending loads, which can readily induce excessive plastic deformation, localized buckling, and fracture failure [2]. Historical earthquakes have demonstrated that a substantial number of pipelines have failed due to fault displacement [3–5]. Investigating the mechanical response and failure mechanisms of pipelines is of profound theoretical and engineering significance for pipeline seismic design and safety evaluation. Polyethylene (PE) pipelines are extensively utilized in urban gas distribution systems owing to their superior corrosion resistance, flexibility, lightweight nature, and ease of installation [6]. In North America, PE pipelines constitute over 90% of newly constructed gas pipeline networks [7]. However, pure PE pipelines exhibit relatively low strength and stiffness, making them susceptible to excessive deformation and failure under extreme external loads [8]. Strain levels under fault displacements can readily surpass critical thresholds, leading to failure [9]. Zhang et al. [10] elucidated the mechanical behavior and failure mechanisms, noting that axial strain distribution follows an “S” pattern, with the cross-sectional flattening parameter serving as a pivotal metric for damage assessment. Weerasekara and Wijewickreme [11] observed that the load-displacement response of buried MDPE pipelines exhibits pronounced nonlinearity and rate dependence. The failure modes of PE pipelines encompass ductile, quasi-brittle, and brittle failures, with material aging during prolonged service exacerbating the degradation of load-bearing capacity [12].To address the performance limitations of pure PE pipelines, steel-reinforced polyethylene (SRPE) pipelines have been developed. By integrating a high-strength steel wire mesh frame within the PE matrix, SRPE pipelines leverage the complementary strengths of both materials—PE provides corrosion resistance and flexibility, while the steel frame enhances strength and stiffness [13]. SRPE pipelines thus exhibit both pressure resistance and corrosion resistance, presenting broad application prospects in oil and gas transportation, municipal water supply and drainage, and other fields [14]. Nevertheless, the composite structure of SRPE pipelines renders their mechanical behavior significantly more complex than that of monolithic materials, particularly under extreme loading conditions such as fault displacements. The synergistic interaction, damage progression, and failure mechanisms between the steel frame and PE matrix remain poorly understood [15]. Recent research indicates that SRPE pipeline failures often initiate with the degradation of the hot-melt adhesive layer, subsequently leading to corrosion and fracture of the steel wires, ultimately resulting in pipeline leakage [16]. Therefore, there is an urgent need to conduct systematic research on the behavior of SRPE pipelines under fault conditions to provide a theoretical foundation for their engineering applications.

Scholars both domestically and internationally have conducted extensive research on the mechanical behavior of buried pipelines under fault action. Newmark and Hall [17] were the first to propose a simplified analytical method, treating pipelines as deformable cables to analyze their strain response. Subsequently, Kennedy et al. [18] and Wang et al. [19]considered the effects of pipeline bending stiffness and soil lateral resistance, respectively, and proposed improved analytical models. With the development of computational mechanics, the finite element method has become a powerful tool for studying this issue. Vazouras et al. [20–22] simulated pipelines using four-node reduced-integration shell elements and systematically investigated the effects of soil and pipeline geometric parameters on structural performance. Xie et al. [23] performed experimental and numerical studies on the mechanical behavior of high-density polyethylene pipelines under combined bending and compression induced by seismic faults, and good consistency was obtained between numerical and experimental results. Zhang [24] analyzed the failure characteristics of buried pipelines and identified shell buckling as the dominant failure mode. Vazouras et al. [25] derived a closed-form force–displacement solution for buried pipelines in tension and incorporated it into refined finite element models through nonlinear spring elements. Li et al. [26] numerically studied the dynamic response of polyethylene pipelines and demonstrated that ground movement rate significantly affects the stress–strain behavior of PE materials. Regarding material aging and structural reliability, Antonelli [27] proposed a reliability-based method to evaluate pipeline resilience by considering material degradation and the interaction of potential failures. Mina [28] numerically investigated the restoring force of pipelines under combined seismic and thermal loads, indicating that loading path governs structural resilience with temperature-dependent material properties considered. Yazdi [29] introduced a probabilistic approach to assess the resilience of subsea pipelines subjected to microbiologically influenced corrosion.

In summary, existing research on the mechanical behavior of buried pipelines under faulting primarily focuses on single-material steel or pure PE pipelines, with systematic studies on SRPE composite pipelines under fault displacements still lacking The composite structure of SRPE pipelines endows them with unique mechanical behaviors. The presence of the steel frame alters the stress transfer path and failure mode of the pipeline, necessitating an in-depth exploration of its inherent mechanism. Based on this, this paper employs the finite element method as the core research tool, aiming to achieve the following research objectives: (1) Establish a refined three-dimensional finite element model for buried SRPE pipelines crossing strike-slip faults and propose a solid modeling method suitable for SRPE pipelines; (2) Systematically study the influence of factors such as the pipe-fault intersection angle, steel frame parameters, and soil type on the mechanical response of SRPE pipelines; (3) Reveal buckling behavior process of SRPE pipelines under strike-slip faulting; (4) Evaluate the applicability of existing critical buckling strain criteria for SRPE pipelines and propose failure criteria considering the influence of fault intersection angle. The research findings can provide a theoretical basis for the seismic design and engineering application of SRPE pipelines.

## 2. Development and validation of the finite element model

### 2.1. Problem definition and geometric model

**Fig 1:**
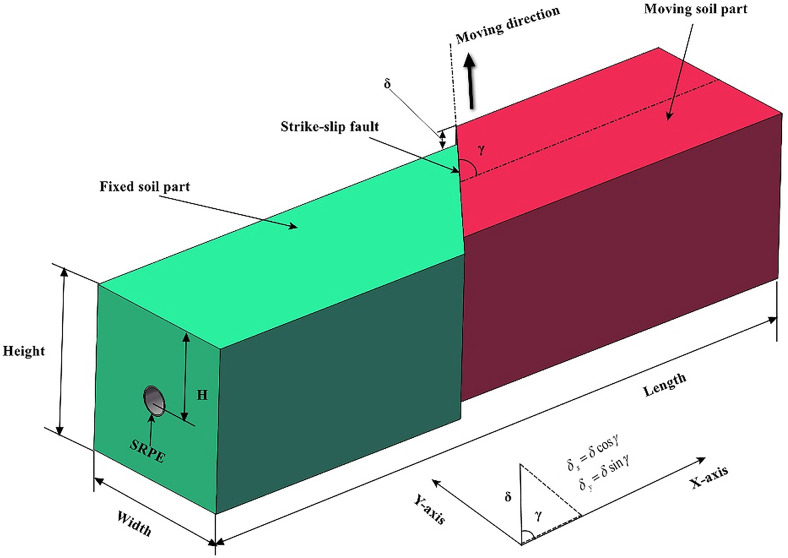
Schematic of a SRPE pipes crossing strike slip fault.

A strike-slip fault refers to a fault type where the two fault blocks slide relatively in the horizontal direction. As shown in Fig 1, when a buried pipeline crosses a strike-slip fault at a certain angle, relative displacement occurs between the two fault blocks in the X−Y horizontal plane. The fault displacement δ can be decomposed into a displacement component δcos(γ) parallel to the pipeline axis and a component δsin(γ) perpendicular to the pipeline axis, where γ is the angle between the pipeline axis and the fault normal direction.

To faithfully characterize the actual mechanical state of buried pipelines subjected to ground dislocation, a high-fidelity three-dimensional finite element model is developed in this study. The model comprises a fixed fault block, a moving fault block, a PE pipe body, and a steel frame. The PE matrix and steel frame are coupled via constraint equations to equivalently represent the SRPE composite pipeline, which is then assembled between the two fault blocks. The relative motion of the fault blocks driven by enforced displacement is employed to exert combined compression and bending loads on the pipeline, thereby systematically revealing the mechanical response of the pipeline. The overall finite element modeling procedure consists of the following five steps: (1) construction of three-dimensional geometric models; (2) definition of boundary conditions and contact interactions; (3) selection of element types and mesh generation; (4) specification of material constitutive models; (5) configuration of analysis steps and solution schemes. To minimize the influence of boundary conditions, the dimensions of the finite element model for the buried SRPE pipeline-soil system are determined according to the following principles: a sufficient length is selected along the pipeline axis (x-direction) to avoid interference from boundary conditions in the large deformation area caused by fault movement [22]. According to the recommendation of Vazouras et al [20,21,30], the axial length of the pipeline should be no less than 60 times the nominal diameter. The parameters of the SRPE pipeline in this study refer to the production execution standard [31]: nominal inner diameter of 100mm, pipe body thickness of 10 mm, steel frame wire diameter of 1.5mm, and wire spacing of 8mm. Considering both calculation accuracy and efficiency, the total length of the pipeline model is selected as L = 18 m (180 times the nominal diameter), with the fault located in the center of the model. The dimensions of the soil model perpendicular to the pipeline axis direction (y-direction) and the vertical direction (z-direction) are both set at 2.2m (approximately 20 times the nominal diameter) to avoid interference from soil boundaries on the mechanical response of the pipeline. The burial depth of the pipeline refers to the “Standard for Design of City Gas Engineering”: the minimum cover thickness under the roadway is ≥0.9m, and that in non-vehicle traffic areas is ≥0.5m. In this study, the depth is set at 1.1 m. This combination of dimensions has been verified through sensitivity analysis to effectively eliminate boundary effects and meet the simulation accuracy requirements.

### 2.2. Boundary conditions and contact algorithms

**Fig 2:**
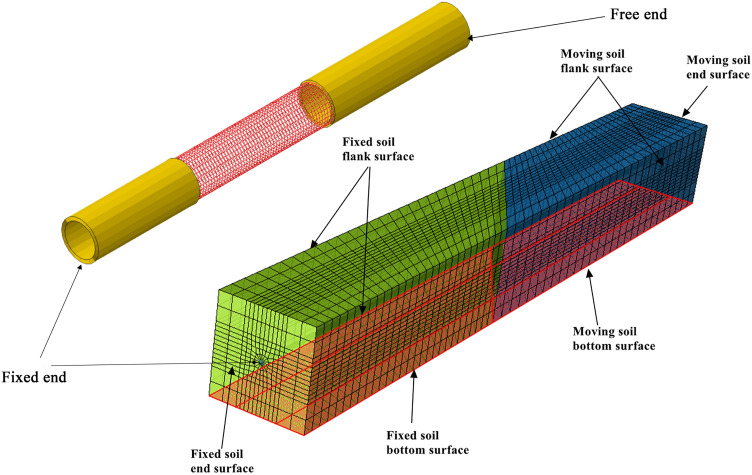
The finite element model of the soil-pipeline system subjected to strike-slip fault movements.

References [6,10,20,22,33], as shown in Fig 2, divide the soil model into a moving fault plate and a fixed fault plate. The moving fault plate moves uniformly within a plane. The boundary conditions and contact methods are reasonable. The PE pipe and the frame are bound and constrained to form a single unit. The bottom, sides, and ends of the moving fault plate move within a horizontal plane, the X-Z plane, until reaching the maximum fault displacement. The bottom, sides, and ends of the fixed fault plate remain fixed and constrained.

The contact surface between the pipe and the soil will change during the solution process, indicating a nonlinear contact between the two. They are set as surface-to-surface contact [21]. Since the stiffness of the pipeline is much greater than that of the soil [22], the contact properties are defined as follows: (1) Normal behavior: “Hard contact” is adopted, allowing separation after contact to simulate the development of the pipe-soil gap. (2) Tangential behavior: The penalty function friction formula is used, with the friction coefficient μ determined based on the type of pipeline coating and soil. For the friction coefficient between the PE pipeline and the sand-soil interface, it is taken to be between 0.45 and 0.7 [32].

### 2.3. Element selection and mesh generation

PE matrix layer: It is simulated using the four-node shell element with reduced integration (S4R) [21]. This element type is suitable for thin shell structure analysis and can accurately capture the bending deformation and local buckling phenomena of the pipeline wall. To accurately simulate the large deformation area near the fault, a non-uniform grid division strategy is adopted: the grid is densified within a range of 5 m on both sides of the fault, with an axial element size of 0.05 m (approximately D/24); a coarser mesh is used in areas away from the fault, with an axial element size of 0.2 m. The circumferential grid is divided into 40 elements, which can meet the convergence requirements after sensitivity verification [31].

Steel frame layer: The steel wires are simulated using beam elements (B31: two-node spatial linear beam element). The geometric parameters such as the composition structure, wire diameter, and spacing of the steel wire mesh frame are determined based on the actual product specifications [33].

Interlayer interaction: In a practical structure, the steel frame is fully embedded within the PE matrix and forms an integrated load-bearing system, analogous to the reinforcement–matrix interaction in reinforced concrete. In the numerical model, tie constraints are adopted to rigidly couple the beam elements representing the steel frame with the shell elements representing the PE matrix. This coupling ensures fully compatible deformation between the two components without relative sliding or separation. Such a constraint allows the steel frame to resist both axial forces and bending moments, enabling accurate representation of the global mechanical behavior of the composite pipeline. Since this study focuses on the macroscopic buckling characteristics and overall deformation patterns of SRPE pipelines, local interfacial mechanics such as debonding and delamination are not considered. Therefore, the use of tie constraints is consistent with the research objectives and conforms to the global deformation mechanism of the pipeline.

### 2.4. Material constitutive model

PE material exhibits pronounced nonlinearity and rate-dependence. In this study In this study, the strike-slip fault is assumed to undergo instantaneous synchronous movement under strong earthquakes, with a total displacement of 400mm, approximately four times the outer diameter of the pipeline. Existing research indicates that fault ruptures induced by typical strong earthquakes generally last 0.5~2s with a relative displacement of 0.2~0.5m. Considering that backfill soil in practical engineering is uniform, well-compacted, and heavily damped, it can effectively buffer the instantaneous slip velocity, delay displacement transfer, and prolong the overall rupture process. Thus, the ultra-rapid slip within 0.5 s is not considered in the model. Based on seismic dynamic characteristics and engineering practice, the total duration of ground dislocation is set to 2 s, and the analysis step time in the finite element simulation is also specified as 2 s. Accordingly, the fault is assumed to move 0.4 m within 2 s., The moving speed is shown in equation (1):


$$\begin{array}{c} v=0.4/2=0.2m/s \end{array}$$ (1)

According to the recommendations of Vazouras et al. [20,21,23], the axial length of the pipeline in the numerical model should be no less than 60 times the nominal diameter. The nominal diameter of the pipeline in this model is 100mm, and thus the effective deformation length is required to be at least 6m. The actual scale of localized deformation near the fault zone is basically within this length range. To conservatively enlarge the affected zone, an additional 2m is adopted as a safety boundary based on the 6mreference length; consequently, the final deformation influence range is determined as $L_{\text{0}}=8m$.

The strain rate of PE material is shown in equation (2):


$$\begin{array}{c} \dot{\varepsilon }=v/L_{\text{0}}=0.2/8=0.025s^{-1} \end{array}$$ (2)

According to Ref. [34], the strain rate of pipelines under seismic loading falls within a reasonable range, and the strain rate adopted in this study is consistent with that reported in Ref. [34]. The Suleiman hyperbolic model is employed to describe the stress-strain relationship of PE material [10], as shown in equation (3):


$$\begin{array}{c} \sigma =\frac{\varepsilon }{a+b\varepsilon } \end{array}$$ (3)

**Fig 3:**
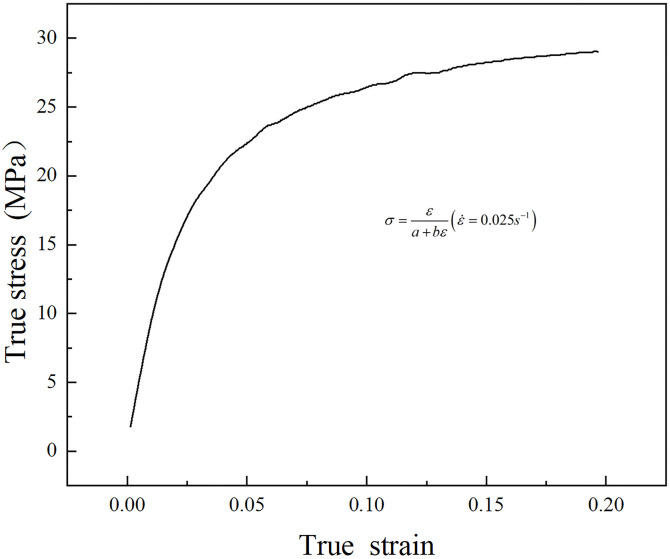
The true stress-strain curve of pipe PE80 $\dot{\varepsilon }=0.025s^{-1}$.

Parameters a and b are strain-rate dependent, which can be calibrated via uniaxial tensile tests conducted at different strain rates. According to Ref.[34], a is set to 0.0007276 and b is set to 0.03859 when the strain rate is on the order of $10^{-2}s^{-1}$. In this work, the strain-rate-dependent elastic modulus method proposed by Reza and Dhar [11] is adopted, and the constitutive model of PE80 pipe material at a strain rate of $0.025s^{-1}$ is employed. This model takes into account the material viscoelasticity and the influence of tensile strain rate. The corresponding mechanical parameters are presented in Table 1 and Fig 3.

**Table 1:** Performance parameter of PE80 pipe.

Material	Density($Kg/m^{3}$)	Elastic modulus E (MPa)	Yield Strength (MPa)	Poisson’s ratio μ	Minimum required strength(MPa)
PE80	951	1512	16.9	0.45	8

The steel frame is constructed using high-strength X80 steel wire. The Ramberg-Osgood model is selected to represent the constitutive model of X80 steel pipeline, and the stress-strain relationship is shown in equation [35]:


$$\begin{array}{c} \varepsilon =\frac{\sigma_{s}}{E}\left[\frac{\sigma }{\sigma_{s}}+\alpha {\left(\frac{\sigma }{\sigma_{s}}\right)}^{N}\right] \end{array}$$ (4)

**Fig 4:**
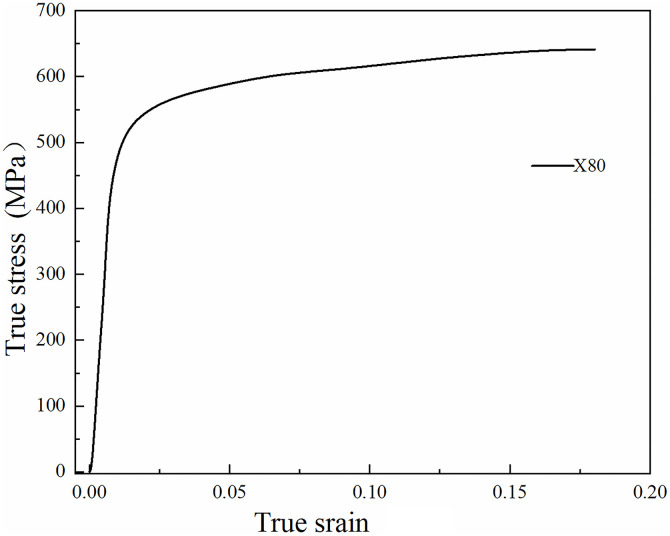
Stress-strain curve of X80 steel pipeline.

In the formula, ε represents the total strain; $\sigma_{s}$ denotes the yield stress, MPa; E signifies the initial elastic modulus; andσstands for stress, with α and N being parameters of the Ramberg-Osgood model. For the X80 steel pipeline utilized in the Second West-to-East Gas Pipeline Project(SWEGPP), $\sigma_{s}=552\;MP\text{a}$, E=207MPa, α=0.86, N=28. The actual stress-strain curve obtained through formula fitting is illustrated in Fig 4 [35].

The Mohr-Coulomb ideal elastoplastic constitutive model has been widely adopted in previous geotechnical numerical studies to characterize the mechanical behaviors of soils [24–26]. With fewer parameters that can be easily calibrated through conventional geotechnical tests, this model well captures the essential shear failure mechanism of geomaterials and exhibits excellent numerical stability and computational convergence. It is highly applicable to common soil types, including sand, clay, and loess, and complies with domestic and international geotechnical design specifications. Currently, the Mohr-Coulomb model remains the most prevalent fundamental constitutive model for numerical simulations of stratum deformation, static loading, and soil-structure interaction. Although this model cannot reproduce the strain hardening, softening, and dilatancy behaviors of soils, these refined mechanical characteristics are not the focus of this study. For the stratum deformation and pipeline mechanical response induced by fault dislocation in this work, the Mohr-Coulomb model can reasonably describe the yield and failure behaviors of soils under monotonic loading, which fully satisfies the accuracy requirements of the present numerical analysis. The parameters involved in the model include: densityρ, elastic modulus E, Poisson’s ratio  μ, internal friction angle φ, cohesion c, and dilatancy angle  ψ. In this study, four typical soil types, namely Cohesive soil, Silty clay, Sandy soil, and Loess, are analyzed. Referring to literature [10], the specific soil parameters are shown in Table 2.

**Table 2:** Mechanical parameters of soil.

Type	Density($Kg/m^{3}$)	Elastic modulus E (MPa)	Poisson’s ratio	Cohesion c (kPa)	Friction angle φ(∘)	Dilation angle ψ(∘)
Cohesive soil	1960	18	0.35	22	34	0
Silty clay	1960	10	0.35	22	25	0
Sandy soil	1800	33	0.44	24.6	11.7	0
Loess	1400	20	0.35	5	15	0

### 2.5. Analysis step setup and solution

Fault displacement is achieved by applying displacement loads on the boundary nodes of the soil mass on one side of the model [36]. Contact pairs are defined on the contact surfaces of the soil masses on both sides of the fault, allowing relative sliding and separation, to simulate the mechanical behavior of the fault fracture surface. The numerical calculation is divided into two analysis steps:

Step 1: In the numerical simulation, the gravitational acceleration is defined as the standard Earth gravity of $\;9\mathrm{.8}m/s^{\text{2}}$, and the duration of the in-situ stress balance step is set to 1 s to fully stabilize the stratum stress and eliminate the initial displacement disturbance caused by model establishment. Meanwhile, a uniform internal pressure load is applied inside the pipeline to accurately reproduce the actual service conditions of the pipeline, providing an engineering-consistent initial stress boundary condition for the subsequent analysis of pipeline mechanical response under fault dislocation.

Step 2: Fault displacement loading. A displacement-controlled loading scheme is adopted in this study, and a strike-slip directional enforced displacement of 400mm is applied to the boundary nodes of the soil mass on one side of the model. The dynamic explicit analysis step is utilized for numerical calculation, and the total loading duration is set to 2s, which is consistent with the fault dislocation time under strong earthquakes assumed in the previous section. Meanwhile, the geometric nonlinearity (Nlgeom) option is activated to fully consider the large deformation effects of pipelines and soils during fault movement, ensuring the authenticity and accuracy of numerical calculation under large deformation conditions.

### 2.6. Model validation

**Fig 5:**
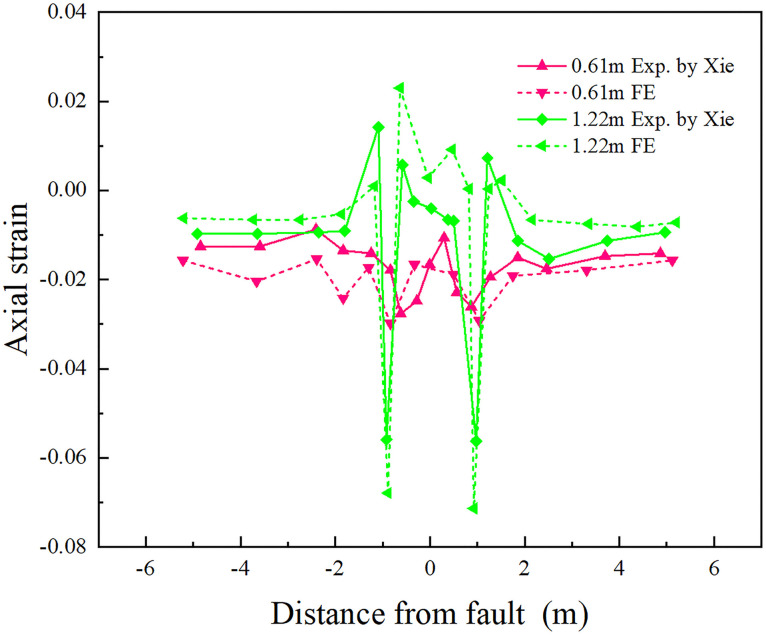
Comparison of the axial strain distributions from the FE simulation with those from the large-scale tests.

Currently, systematic experimental data of SRPE pipelines subjected to strike-slip faults are still limited. In contrast, previous literature [23] has carried out comprehensive experimental and numerical investigations on the mechanical behavior of HDPE pipelines under strike-slip fault displacement, which can provide a credible benchmark for model verification. The numerical modeling frameworks of SRPE and HDPE pipelines are highly consistent. The only structural difference lies in the embedded steel reinforcement frame of SRPE pipelines, while the geometric dimensions, boundary conditions, contact interactions, and loading configurations remain identical. Accordingly, the experimental data reported in Ref. [23] are adopted to validate the accuracy of the proposed numerical model. The HDPE numerical model from the literature is precisely reproduced first to calibrate the mesh scheme, boundary constraints, material parameters, and analysis step settings. After the modeling methodology is fully verified, only the pipeline structural form and corresponding geometric parameters are modified, whereas all boundary conditions, contact attributes, and solving configurations are retained unchanged. This strategy effectively reduces artificial modeling errors and guarantees the computational accuracy of the SRPE pipeline model. The verification is based on large-scale soil-structure interaction tests conducted at Cornell University [23]. The tested pipeline has a total length of 10.56\hspace{0.5em}m, an outer diameter of 0.4m, a wall thickness of 0.024m, and a buried depth of 1.22m, with a fault intersection angle of 65° and a maximum fault displacement of 1.22m. Fig 5 compares the strain distribution at the pipeline buckling section between numerical predictions and experimental measurements under fault displacements of 0.61m and 1.22m. The results indicate that the numerically simulated strain distribution trends agree well with the experimental data [23]. At the fault displacement of 0.61m, the maximum longitudinal compressive strain obtained by simulation is −3.0%, while the experimental value is −2.8%. When the fault displacement reaches 1.22 m, the simulated maximum longitudinal compressive strain is −7.1%, compared with the experimental value of −5.9%. The relative errors between numerical and experimental results are within 10% for all working conditions. The comparison confirms that the established finite element model is reliable, and the adopted modeling and parameter-setting methods are reasonable, which can be further applied to investigate the mechanical responses of SRPE pipelines under strike-slip fault dislocation.

**Fig 6:**
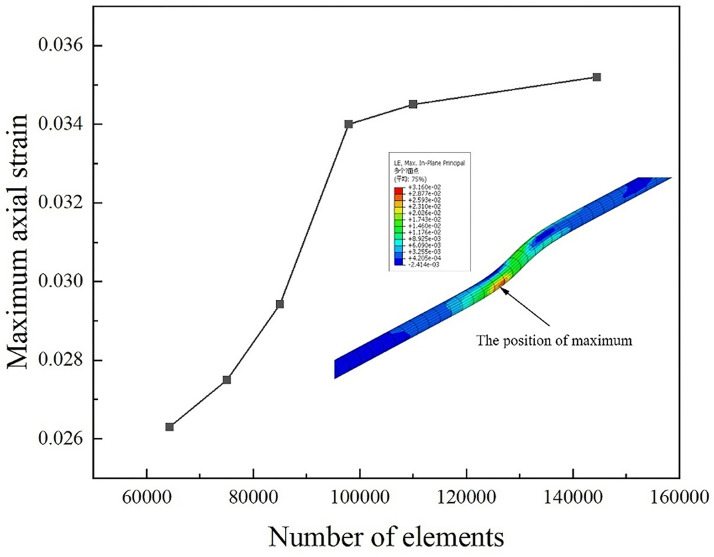
The correlation between the number of grids and the maximum axial strain.

To verify the rationality of the number of elements, a grid sensitivity analysis was conducted as shown in Fig 6 Six grids with different densities were designed. Under the condition of a fault displacement of 2D, the axial strain extremum measured by tension was used as the evaluation index. When the number of model elements was 100,000, the maximum axial strain value tended to be stable. When the number of grids increased from 100,000–150,000, the strain value increased by 2.9%. Considering both computational accuracy and efficiency, the number of grids for finite element analysis was set to 100,000.

The adopted element combination and contact configuration balance computational accuracy and numerical efficiency, which fully satisfies the simulation requirements of complex pipe-soil interaction under strike-slip fault action.

## 3. Results and discussion

### 3.1. Pipe-soil interaction under strike-slip fault action

**Fig 7:**
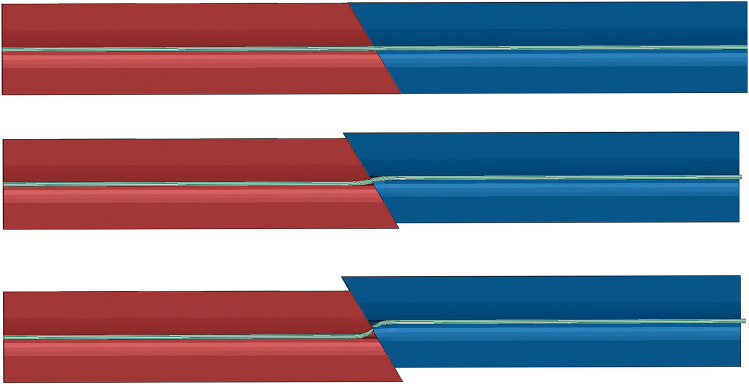
Deformation of pipeline-soil system under strike-slip fault.

Based on the national standard [31], a detailed analysis is conducted by selecting the reference operating conditions: the outer diameter of the pipeline D=100mm, wall thickness t=10mm (diameter-to-thickness ratio D/t=10), steel frame wire diameter of 1.5mm, center-to-center distance of the warp wire of 8mm, burial depth H=1.2m, soil type as Cohesive soil, fault intersection angle $\gamma =60^{\circ }$. Fig 7 illustrates the deformation process of the pipeline-soil interaction under strike-slip faulting: before the stratum dislocation, the backfill soil is in full contact with the surface of the buried pipeline, and the pipeline bears the internal pressure and the force exerted by the surrounding soil; as the fault displacement increases, the pipeline undergoes bending deformation, and the pipe sections on both sides of the fault plane only make contact with the backfill soil on one side. The stratum movement causes the pipeline to bear additional bending moments and frictional forces, and the pipeline deformation exerts a counteracting force on the surrounding soil. When the displacement further increases, local buckling occurs in the pipeline sections at a certain distance on both sides of the fault plane, and the pipe sections no longer form a smooth curve. In summary, during the fault movement process, the buried pipeline does not maintain full contact with the surrounding soil, and there are differences in the pipe-soil contact pressure, leading to uneven distribution of frictional forces on the pipe sections. Therefore, using an ideal pipe-soil frictional force calculation model is not appropriate.

**Fig 8:**
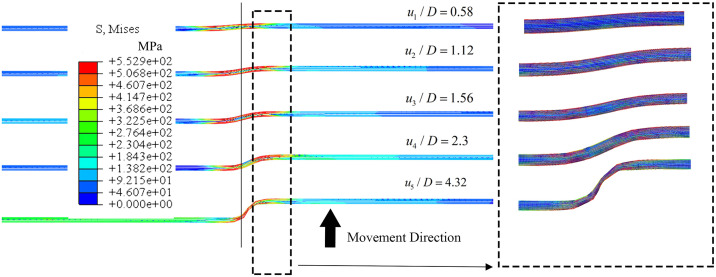
Distribution contour of von Mises stress along the tube at different displacement values.

The frame is located inside the PE pipeline. For visualization purposes, the steel frame is depicted externally to the PE pipe in the figures. Based on the finite element numerical simulation results shown in Fig 8, a systematic analysis was conducted on the evolution law of Mises stress in the buckling region section with non-dimensional fault displacement ($u_{i\;}/D$) under the action of fault movement on buried pipelines. This revealed the mechanical response mechanism of pipelines from elastic deformation to plastic buckling, and then to large deformation instability. The von Mises stress contour plot in the figure shows that the evolution of the pipeline stress field exhibits significant phased characteristics, which are directly related to the level of fault displacement. In the small displacement stage ($u_{1\;}/D=0.58\text{,}u_{\text{2}}\;/D=1.12$), the pipeline is in the initial state of elastic buckling, and Mises stress forms a localized concentration zone only near the fault displacement surface, with the maximum stress value reaching 552.9 MPa, which is close to the yield strength of the pipeline material. The stress level in the pipeline area away from the displacement surface is extremely low, and the overall deformation is dominated by elastic bending, without large-scale plastic deformation. At this time, the stress along the pipeline axis exhibits a typical “local high stress gradient distribution,” with the displacement surface being the core of stress concentration, reflecting the initial mechanical process of fault forced displacement transmitted to the pipeline through pipe-soil interaction. In the medium displacement stage ($u_{\text{3}}\;/D=1.56\text{,}u_{\text{4}}\;/D=2.3$), the pipeline enters the plastic buckling development stage, and the high stress area continues to expand along the pipeline axis to both sides. The peak stress area shifts from near the displacement surface to the buckling wave crest and trough positions, and the section stress distribution transitions from localized concentration to continuous distribution along the buckling deformation zone. The pipeline undergoes irreversible plastic deformation, and the buckling morphology transitions from initial corrugated shape to large curvature bending, reflecting the coupled evolution mechanism of “displacement drive-buckling development-stress redistribution.” In the large displacement stage $\text{(}u_{\text{5}}\;/D=4.32)$, the pipeline enters the buckling instability and large deformation limit state, and von Mises stress reaches full-field saturation at the buckling core section. The high stress area completely covers the entire buckling section, and the pipeline undergoes significant section distortion and large plastic deformation. The stress distribution exhibits the characteristic of “high stress in the full section of the buckling core area + low stress in the distal elastic area.” The pipeline bearing capacity enters the plastic limit state, at which point buckling deformation and stress concentration form positive feedback, further exacerbating pipeline deformation.

### 3.2. Stress-strain distribution patterns along the pipeline

**Fig 9:**
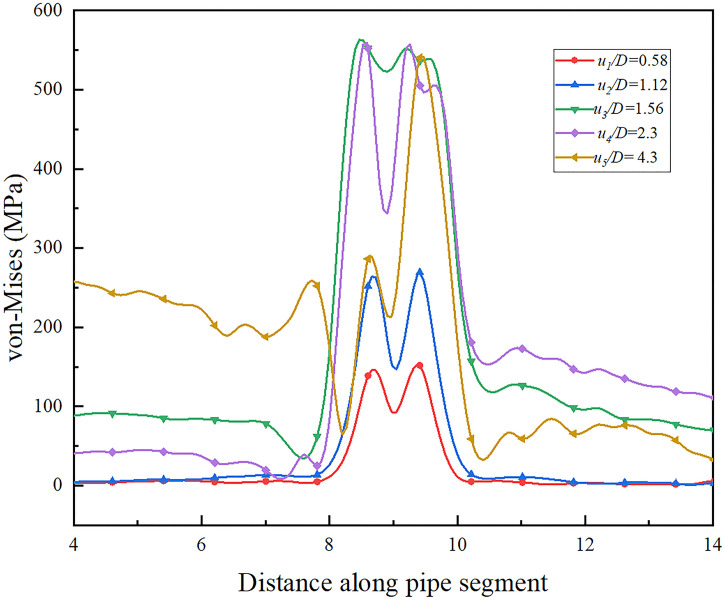
Distribution curve of von Mises stress along the pipe length at different displacements.

**Fig 10:**
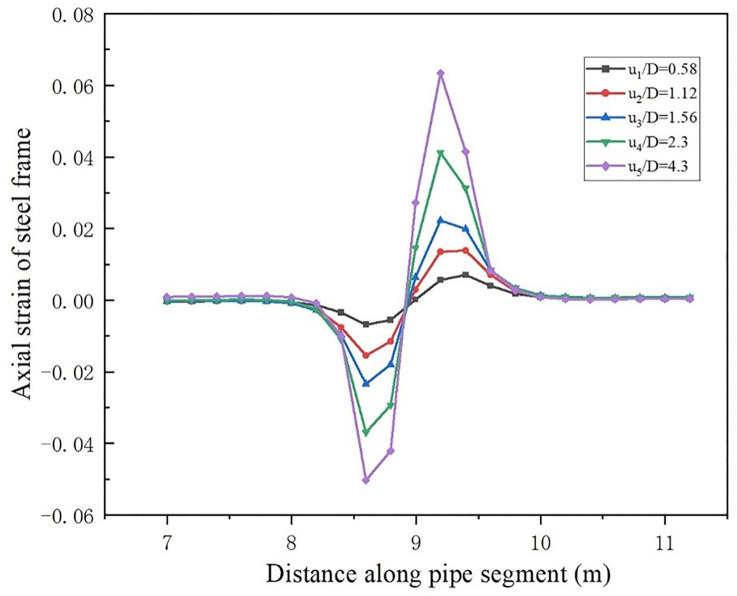
Axial strain curve of steel frame distributed along the pipeline at different displacements.

Fig 9 and Fig 10 systematically demonstrate the distribution patterns of von Mises stress and axial strain along the pipeline axis under the influence of fault movement in the SRPE pipeline steel frame. They reveal the mechanical response evolution mechanism and buckling instability characteristics of the pipeline under different non-dimensional fault displacements ($u_{i}\;/D$). As shown in Fig. 9 (von Mises stress distribution), the von Mises stress of the steel frame exhibits a significant local concentration distribution along the pipeline axis. The stress peak areas strictly correspond to the vicinity of the fault movement plane (approximately within the range of 8~10m). Furthermore, the stress level and distribution range exhibit phased evolution characteristics with the increase of fault displacement: under small displacement conditions ($u_{1\;}/D=0.58\text{,}u_{\text{2}}\;/D=1.12$), the stress concentration areas are relatively narrow, with peak stresses of approximately 150 MPa and 270 MPa, respectively. The stress levels in distant pipelines (<8m,>10mm regions) are extremely low (<50 MPa), and the pipeline as a whole is in the elastic deformation stage, with local bending stress concentration only occurring near the movement plane. Under medium displacement conditions ($u_{\text{3}}\;/D=1.56\text{,}u_{\text{4}}\;/D=2.3$), the stress concentration areas significantly expand axially to both sides, and the peak stresses rapidly climb to over 550MPa, approaching the yield strength of steel. The stress levels in the far-field pipelines rise simultaneously, and the pipeline enters the plastic buckling development stage. The stress distribution evolves from local concentration to continuous high-stress zones. Under large displacement conditions ($u_{\text{5}}\;/D=4.3$), the peak stress areas cover the entire movement segment, and the peak stresses remain at a yield plateau of around 550MPa. The stress levels in distant pipelines (4 8m,10 14mregions) continue to rise to over 200\hspace{0.5em}MPa, and the pipeline undergoes global plastic large deformation, with the buckling instability pattern fully formed. The stress distribution exhibits characteristics of “core area saturation and continuous evolution in the far field.”

Combining the evolution law of axial strain distribution in Fig 10, the coupling response mechanism of stress-strain can be further revealed: the axial strain exhibits a symmetrical tension-compression bimodal distribution along the pipeline axis, forming a compression strain valley (at about 8.5m) and a tension strain peak (at about 9.2m) near the fault plane. The strain amplitude is positively correlated with the fault displacement. Under small displacement conditions, the strain amplitude is extremely small (maximum tensile strain <0.01, maximum compressive strain <0.01), and the pipeline mainly deforms through elastic bending, with a narrow range of tension-compression strain zones. Under medium displacement conditions, the amplitudes of tensile and compressive strains rapidly increase, with the maximum tensile strain reaching 0.04 and the maximum compressive strain reaching 0.04. The tension-compression strain zones significantly expand along the axis, and the pipeline undergoes irreversible plastic deformation, with the buckling morphology transitioning from initial corrugated shape to large curvature bending. Under large displacement conditions, the strain amplitude reaches the limit state, with the maximum tensile strain climbing to 0.065 and the maximum compressive strain reaching −0.055. The tension-compression strain zones completely cover the fault displacement segment, fully corresponding to the high stress region of Mises stress throughout the domain, verifying the mechanical essence that fault displacement drives the pipeline to undergo bending-tension composite deformation through forced displacement, thereby inducing stress concentration and plastic buckling.

### 3.3. Synergistic mechanism of steel frame and PE matrix

**Fig 11:**
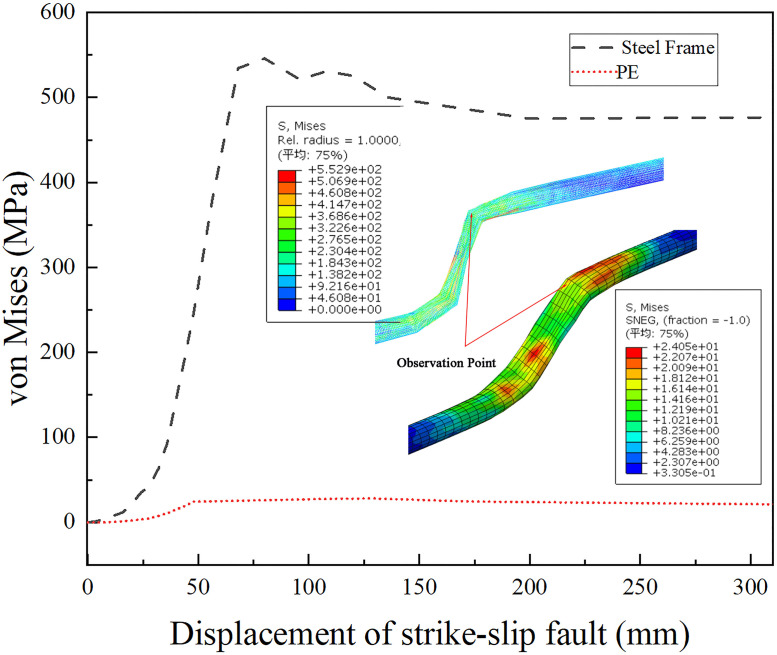
von Mises stress responses of steel frame and PE matrix of SRPE pipeline under 90° strike-slip fault displacement.

**Fig 12:**
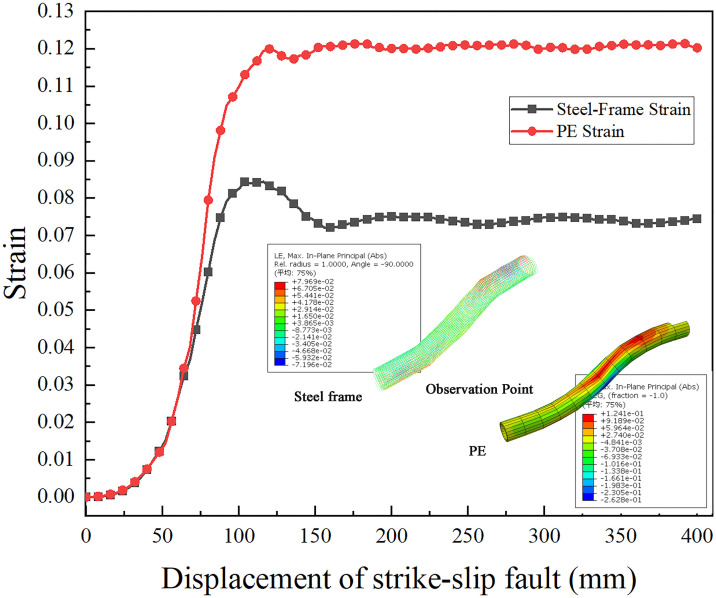
Strain responses of steel frame and PE matrix of SRPE pipeline under 90° strike-slip fault displacement.

The Fig 11, Fig 12 compares the stress and strain responses of the steel frame and PE matrix of SRPE pipelines under different fault displacements at a 90° strike-slip angle, revealing the cooperative working mechanism between the two structural components. At the initial loading stage (0~60mm), the steel frame and PE matrix exhibit nearly consistent growth rates of stress and strain, and the pipeline undergoes coordinated elastic deformation with uniform stress distribution. When the fault displacement increases to 60~120mm, the PE matrix firstly enters the plastic deformation stage. Its strain remains at a relatively high level while the von Mises stress is maintained at a low state, which primarily functions to transfer deformation and dissipate energy. In contrast, the steel frame begins to bear the major external load, with its stress and strain rising rapidly to the peak value. At the late loading stage (displacement> 120mm), the steel frame reaches a stable post-yield state and undertakes most of the combined axial tension and bending loads. Meanwhile, the PE matrix maintains a low-stress and high-deformation state, continuously restraining the local buckling of the steel frame. The dynamic load redistribution process indicates that an efficient cooperative bearing system characterized by “steel frame bearing load and PE matrix providing constraint and energy dissipation” is formed inside the SRPE pipeline. This structural cooperation significantly improves the ultimate deformation capacity and anti-buckling performance of SRPE pipelines under strike-slip fault displacement.

### 3.4. Influence of intersection angle on critical displacement

**Fig 13:**
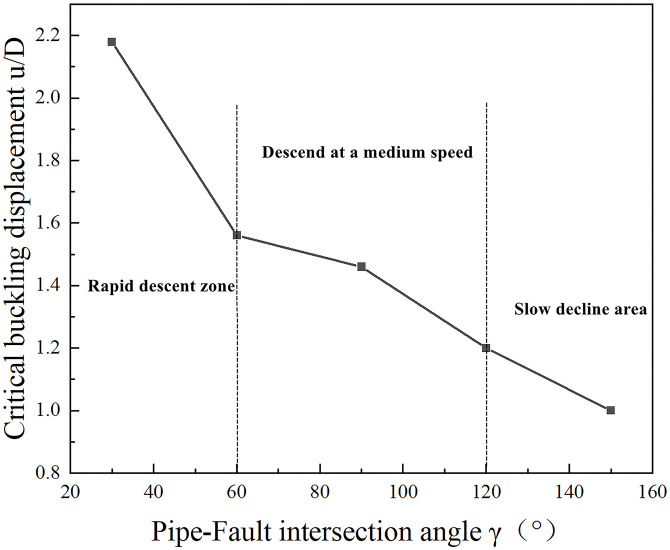
Relationship curve between critical displacement u/D and Pipe-Fault intersection angle γ.

From Fig 13, it can be observed that the critical buckling displacement exhibits a significant nonlinear negative correlation with the increase in intersection angle. The overall attenuation characteristics can be divided into three distinct stages, which profoundly reflect the transformation of the load application mode: the rapid attenuation zone ($\gamma <60^{\circ }$): this region corresponds to the geometric state where the pipeline intersects the fault at a small acute angle. In this state, the fault movement acts almost entirely along the pipeline axis, with extremely high load transfer efficiency. The medium-speed attenuation zone ($60^{\circ }\leq \gamma <120^{\circ }$): as the angle increases, the axial component of the movement load gradually weakens, and the lateral bending component begins to increase in proportion. The interaction mode between the pipeline and the fault transitions from “axial cutting” to “bending and compression.” In this stage, the synergistic bearing mechanism between the steel frame and the PE matrix begins to play a key role, dissipating some of the movement energy through deformation coordination, thereby slowing down the decrease rate of the critical displacement. The slow-speed attenuation zone ($\gamma \geq 120^{\circ }$): this region corresponds to the geometric state where the pipeline is nearly perpendicular to the fault. Currently, the fault movement mainly produces lateral shear, and the pipeline resists external loads through bending deformation. Due to the relatively strong lateral constraint perpendicular to the fault strike and the effective restriction of section distortion by the rigid support of the steel frame, the critical buckling displacement remains at a relatively low but stable level, no longer decreasing sharply. The above principle clarifies that the larger the angle (closer to perpendicular), the less likely the pipeline is to be “cut off” by the fault, and the stronger its resistance to movement; the smaller the angle (more parallel), the more susceptible the pipeline is to axial traction damage.

### 3.5. Influence of intersection angle on buckling morphology and location

The figure illustrates the displacement of the same 4 times the pipe diameter under the action of strike-slip faults, as well as the contour plots of the maximum plane principal strain (absolute value) and buckling morphology of buried pipelines at different pipeline-fault intersection angles γ (30°, 60°, 90°, 120°, 150°). The dashed line represents the soil-soil contact surface (fault displacement surface), and the arrows indicate the range of the fault influence zone.

(1) Deformation contour plot of the pipeline at $\gamma =30^{\circ }$

**Fig 14:**
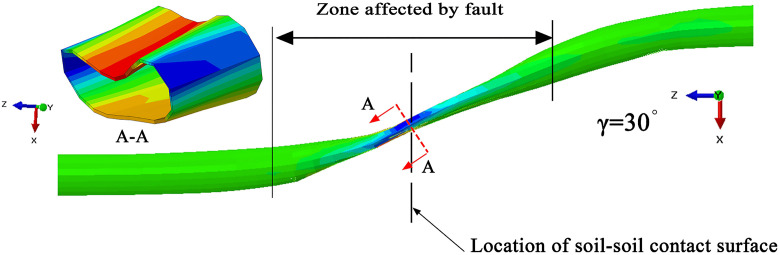
Deformation contour plot of the pipeline at $\gamma =30^{\circ }$.

As shown in Fig 14, the overall deformation of the pipeline is gradual, with the pipeline being uniformly flattened along the affected area. The upper part of the cross-section deforms more than the lower part, indicating that the upper part undergoes more compression deformation.

(2) Deformation contour plot of the pipeline at $\gamma =60^{\circ }$

**Fig 15:**
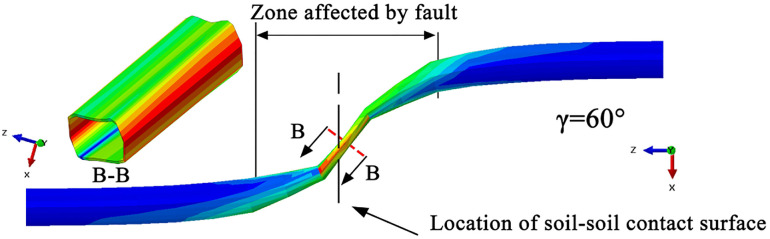
Deformation contour plot of the pipeline at $\gamma =60^{\circ }$.

As shown in Fig 15, the pipeline exhibits significant bending deformation near the fault displacement surface, forming a clear “single wave” bend. The range of the fault-affected area narrows, the degree of deformation concentration increases significantly, the deviation of the pipeline axis from the original straight-line increases, and the pipeline section undergoes obvious elliptical deformation.

(3) Deformation contour plot of the pipeline at $\gamma =30^{\circ }$

**Fig 16:**
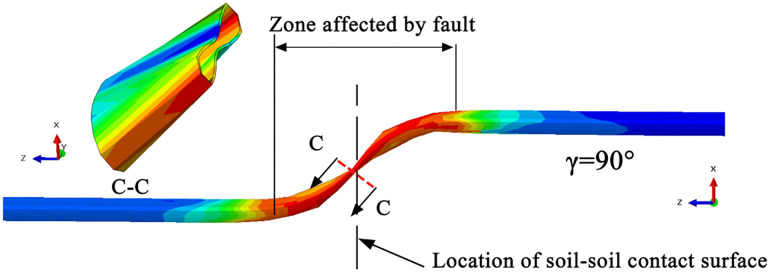
Deformation contour plot of the pipeline at $\gamma =90^{\circ }$.

As shown in Fig 16, the maximum principal strain level of the pipeline at the fault displacement surface reaches the medium-high range. The high strain area is completely concentrated at the bend of the fault displacement surface, with a significant strain gradient on both the inner and outer sides of the section. Severe plastic deformation has occurred in local areas, and local buckling has progressed to a fully developed stage. The pipeline section has undergone severe non-circular deformation.

(4) Deformation contour plot of the pipeline at $\gamma =120^{\circ }$

**Fig 17:**
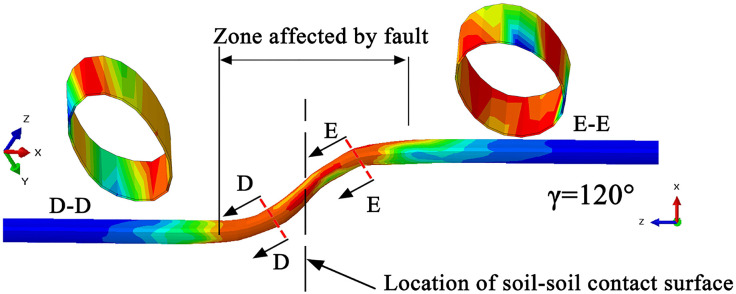
Deformation contour plot of the pipeline at $\gamma =120^{\circ }$.

As shown in Fig 17, the bending morphology of the pipeline exhibits a “gentle S-shape,” with a slightly reduced degree of deformation concentration, but still significantly higher than that under small-angle conditions. The range of the fault influence zone has slightly expanded, and the location of local buckling has shifted towards the side away from the fault plane. The degree of pipe wall sagging has eased compared to the $\gamma =90^{\circ }$ condition, but the buckling morphology is more complex, with local buckling evolving from a “single-wave type” to a “multi-wave type.”

(5) Deformation contour plot of the pipeline at $\gamma =150^{\circ }$

**Fig 18:**
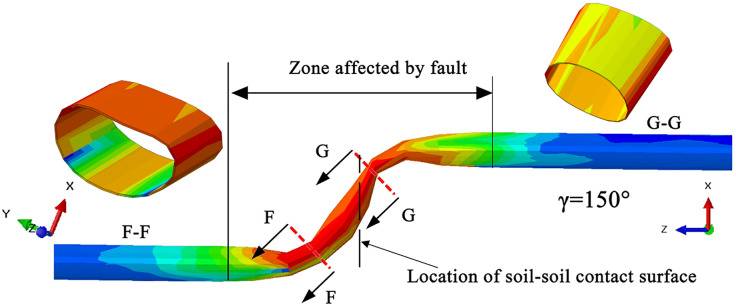
Deformation contour plot of the pipeline at $\gamma =150^{\circ }$.

As shown in Fig 18, two relatively concentrated and extremely severe local bending limit states are formed at the fault displacement surface of the pipeline. The maximum principal strain (absolute value) reaches its peak, severe plastic damage and geometric instability occur in local areas, and local buckling fully develops and enters the post-buckling stage.

The γ angle significantly alters the stress distribution characteristics of the pipeline cross-section. At a small γ angle of 30°, axial tensile stress predominates, with minimal stress gradients on the inner and outer sides, and no obvious stress concentration. At $\gamma =60^{\circ }$, the bending effect intensifies, the difference between compressive stress on the inner side of the cross-section and tensile stress on the outer side increases, and the stress gradient begins to emerge. Under pure bending at $\gamma =90^{\circ }$ the stress gradient of the cross-section reaches its peak, with high stress concentration in the inner compression zone and simultaneous increase in stress in the outer tension zone. At $\;\gamma =120^{\circ }$, the coupling of bending and compression leads to a multi-peak distribution of stress in the cross-section, with multiple high stress concentration points on the compression side, resulting in a more complex stress distribution. At $\gamma =150^{\circ }$, local compression causes the cross-sectional stress to exhibit extreme concentration, with a sharp increase in compressive stress in the buckling and wrinkling region, and stress relaxation and necking characteristics on the tension side due to large deformation, resulting in an extremely uneven overall stress distribution and the highest risk of failure. The γangle determines the stress mode of the pipeline by changing the proportion of load components due to fault movement: when $\gamma <90^{\circ }$, the “tension-bending coupling” predominates, while when $\gamma <90^{\circ }$, the “bending-compression coupling” predominates, ultimately leading to the evolution of buckling morphology from “uniform deformation” to “local extreme instability.”

### 3.6. Enhancement effect of steel frame

**Fig 19:**
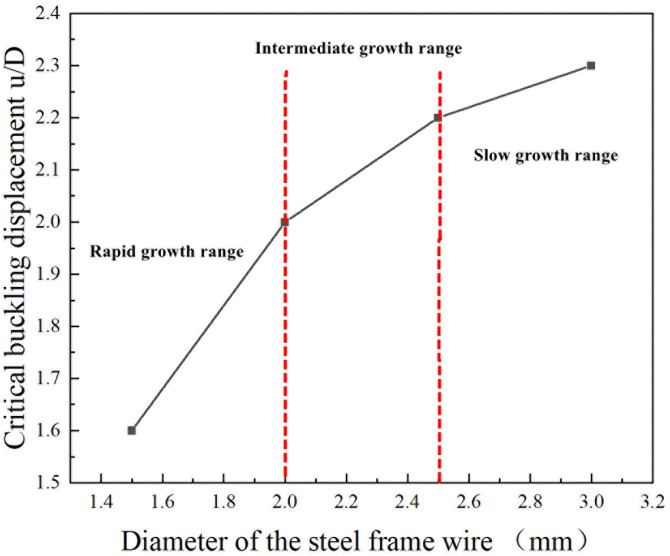
Relationship curve between critical buckling displacement u/D and steel frame wire diameter.

Fig 19 systematically reveals the evolutionary law of dimensionless critical buckling displacement (u/D) of buried SRPE pipelines with respect to steel wire diameter in the steel frame, directly reflecting the reinforcing effect of steel frame diameter on pipeline resistance to fault-dislocation-induced buckling. Results demonstrate a significant positive correlation between critical buckling displacement and steel wire diameter: as wire diameter increases, pipeline’s ultimate load-bearing capacity against fault-dislocation buckling continuously improves, exhibiting distinct three-stage differentiation in reinforcing effect. Quantitative reinforcing effects per stage: 1. Rapid Growth Zone (Wire Diameter d<2.0\hspace{0.33em}mm): Critical buckling displacement shows sharp linear increase with wire diameter, surging from u/D=1.60 at d=1.5\hspace{0.33em}mm to u/D=2.00 at d=2.0\hspace{0.33em}mm—absolute increase 0.40, relative increase 25.0%, averaging ~5.0% critical displacement growth per 0.1mm wire diameter increase. With small steel frame diameter, pipeline stiffness is PE matrix-dominated; small wire diameter increases significantly enhance axial/bending stiffness, yielding most pronounced critical displacement improvement and high parameter sensitivity. 2. Moderate Growth Zone (2.0\hspace{0.33em}mm≤d<2.5\hspace{0.33em}mm): Critical displacement growth rate markedly decelerates as wire diameter further increases, with u/D steadily rising from 2.00 to 2.20—absolute increase 0.20, relative increase 10.0%, averaging ~2.0% growth per 0.1 0.1mm wire diameter increase. Steel frame becomes core load-bearing component, synergistic bearing mechanism between PE matrix and steel frame fully activated; stiffness gains from wire diameter increase are gradually buffered by matrix deformation coordination, shifting reinforcing effect from “stiffness-dominated” to “synergistic bearing-dominated” and stabilizing growth rate. 3. Slow Growth Zone (d≥2.5\hspace{0.33em}mm): When wire diameter exceeds 2.5mm, critical displacement growth further slows, entering slow-growth plateau. At d=3.0\hspace{0.33em}mm, u/D only reaches 2.30—absolute increase 0.10, relative increase 4.5%, averaging ~0.9% growth per 0.1mmwire diameter increase. Steel frame stiffness is sufficiently high; buckling resistance improvement bottleneck shifts from steel frame strength to PE matrix ductility and interfacial bonding performance. Further wire diameter increases yield diminishing marginal load-bearing capacity gains, approaching reinforcing effect saturation. From full-cycle enhancement perspective, using d=1.5\hspace{0.33em}mm as baseline, wire diameter increase to 3.0mm achieves 43.75% total relative increase in SRPE pipeline critical buckling displacement—empirically validating steel frame diameter’s significant reinforcing effect on buckling resistance.

### 3.7. Influence of soil type and stiffness

**Fig 20:**
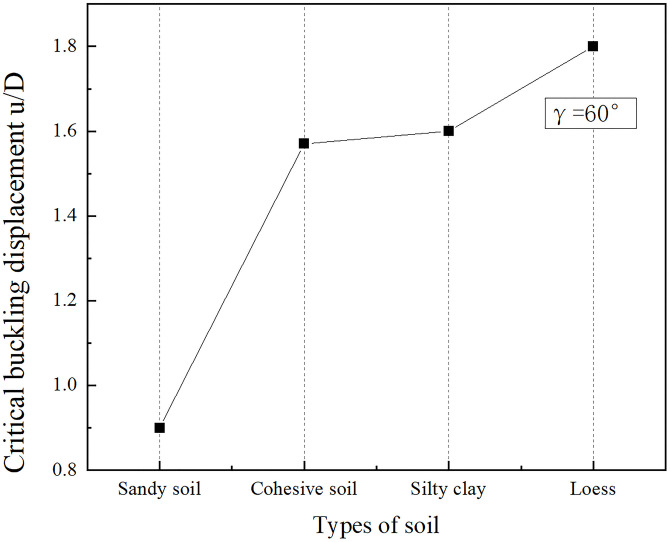
Relationship curve between soil type and critical buckling displacement.

Fig 20 systematically reveals the evolution of dimensionless critical buckling displacement u/D of buried SRPE pipelines with backfill soil types under the condition of pipeline-soil fault intersection angle γ = 60°. It quantifies the influence of different soil mechanical properties on the anti-fault-dislocation buckling performance of the pipeline. The results indicate that the critical buckling displacement of the pipeline increases in a stepwise manner with the enhancement of soil cohesion and stiffness. Significant differences in anti-buckling performance exist under different soil conditions, with quantitative enhancement effects as follows:

Using sandy soil (u/D=0.90) as the baseline condition, the critical displacement and relative increases for each soil type are as follows: Sandy soil: critical buckling displacement u/D=0.90, the lowest value among all conditions, indicating the weakest anti-buckling performance. Cohesive soil: critical displacement u/D=1.57, with an absolute increase of 0.67 and a relative increase of 74.4% compared to sandy soil, achieving a leap in anti-buckling capability. Silty clay: critical displacement u/D=1.60, with an absolute increase of 0.70 and a relative increase of 77.8% compared to sandy soil; however, the absolute increase compared to cohesive soil is only 0.03, with a relative increase of 1.9%, indicating a flattening of the enhancement effect. Loess: critical displacement u/D=1.80, with an absolute increase of 0.90 and a relative increase of 100.0% compared to sandy soil, the highest among all conditions, representing optimal anti-buckling performance; compared to silty clay, the absolute increase is 0.20 with a relative increase of 12.5%, achieving further improvement. The mechanical essence of these patterns lies in the cohesion, internal friction angle, and stiffness characteristics of different soils, which directly determine the constraint strength and load transfer efficiency of soil-pipe interactions. Buried pipelines in sandy soil strata exhibit a higher risk of buckling failure compared with those in clayey soil strata. The cohesion effect of clay enhances the structural integrity and lateral restraint capacity of the stratum, and the improved soil stiffness provides sufficient passive earth resistance to constrain the compressive deformation of pipelines. In contrast, sandy soil transfers force merely through inter-particle friction with weak overall restraint performance. Voiding easily occurs around the pipeline during stratum movement, which weakens the confinement effect and allows unconstrained free deformation of the pipeline’s compressive section, eventually developing into progressive buckling failure. Moreover, the influence discrepancy of different soil layers varies with loading conditions. The difference in pipeline deformation restraint between sandy and clayey soils is insignificant under uniform settlement. Nevertheless, under large-displacement intense disturbances such as fault dislocation and lateral extrusion, pipelines buried in sandy soil are far more susceptible to buckling instability due to the insufficient stratum confinement.

### 3.8. Influence of intersection angle on critical buckling strain

The figure compares the correlation between the critical buckling strain of SRPE pipelines and classical critical strain criteria under various strike-slip fault angles, revealing the influence law of fault angle on the pipeline buckling failure control mechanism and characterizing the mechanical evolution within the angle range of 30°–150°. In the figure, the solid lines represent the critical tensile strain (CTS), while the dashed lines denote the critical compressive strain (CSC). Three mainstream design specifications are adopted as the judging criteria in this study, including the Canadian oil and gas pipeline standard CSA Z662-2023, the European buried plastic composite pipeline standard EN 13476−3, and the Chinese oil and gas transmission pipeline design code GB 50470−2017. These specifications are all applicable to geological disasters such as reverse fault dislocation, strike-slip fault movement, and strong seismic displacement, adopting the global deformation strain of pipelines as the core failure index. The critical tensile and compressive strain thresholds of the three standards are symmetrically defined as ±0.075, ± 0.08, and ±0.01, respectively. The numerical results indicate that pipelines are dominated by tensile stress under small-angle strike-slip fault excitation of 30°. The critical tensile buckling strain significantly exceeds the tensile limit specified by current codes, demonstrating that the buckling failure under small-angle fault dislocation is mainly controlled by tensile deformation, and the actual ultimate tensile capacity of the pipeline is higher than the code limitation. As the fault angle increases gradually from 30° to 150°, the dominant mechanical response of the pipeline transforms steadily, with the tensile effect weakening and the compressive effect strengthening continuously. Accordingly, the buckling control mechanism transitions progressively from tension-dominated to compression-dominated. Under the large-angle condition of 150°, the pipeline is mainly subjected to compressive deformation, and its critical compressive buckling strain far exceeds the code-specified compressive threshold, which becomes the primary factor inducing pipeline buckling failure. Overall, the variation of fault angles from 30° to 150° leads to distinct tensile and compressive mechanical responses and strain over-limit characteristics of SRPE pipelines. The safety margin against code criteria and failure control mechanisms differ significantly under different fault angles. The findings can provide a theoretical basis for buckling safety evaluation and engineering protection of SRPE pipelines under diverse strike-slip fault conditions.

**Fig 21:**
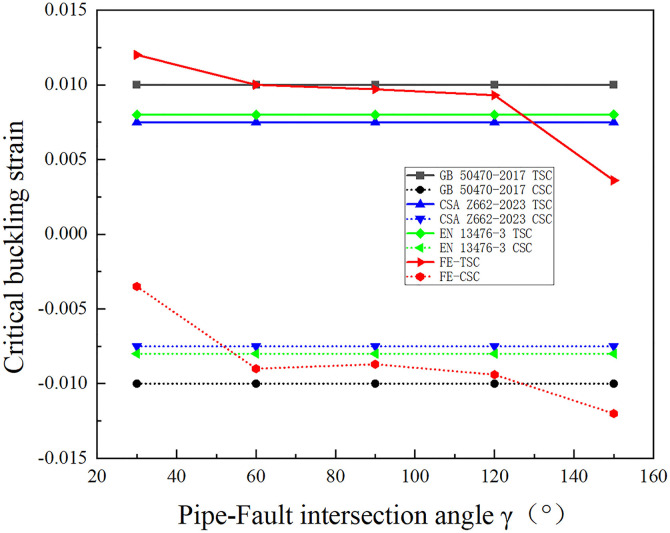
Relationship curve between Pipe-Fault intersection angle γ and critical buckling strain.

The Fig 21 compares the correlation between the critical buckling strain of SRPE pipelines and classical critical strain criteria under various strike-slip fault angles, revealing the influence law of fault angle on the pipeline buckling failure control mechanism and characterizing the mechanical evolution within the angle range of 30°–150°. In the figure, the solid lines represent the critical tensile strain (CTS), while the dashed lines denote the critical compressive strain (CSC). Three mainstream design specifications are adopted as the judging criteria in this study, including the Canadian oil and gas pipeline standard CSA Z662-2023, the European buried plastic composite pipeline standard EN 13476−3, and the Chinese oil and gas transmission pipeline design code GB 50470−2017. These specifications are all applicable to geological disasters such as reverse fault dislocation, strike-slip fault movement, and strong seismic displacement, adopting the global deformation strain of pipelines as the core failure index. The critical tensile and compressive strain thresholds of the three standards are symmetrically defined as ±0.075, ± 0.08, and ±0.01, respectively. The numerical results indicate that pipelines are dominated by tensile stress under small-angle strike-slip fault excitation of 30°. The critical tensile buckling strain significantly exceeds the tensile limit specified by current codes, demonstrating that the buckling failure under small-angle fault dislocation is mainly controlled by tensile deformation, and the actual ultimate tensile capacity of the pipeline is higher than the code limitation. As the fault angle increases gradually from 30° to 150°, the dominant mechanical response of the pipeline transforms steadily, with the tensile effect weakening and the compressive effect strengthening continuously. Accordingly, the buckling control mechanism transitions progressively from tension-dominated to compression-dominated. Under the large-angle condition of 150°, the pipeline is mainly subjected to compressive deformation, and its critical compressive buckling strain far exceeds the code-specified compressive threshold, which becomes the primary factor inducing pipeline buckling failure. Overall, the variation of fault angles from 30° to 150° leads to distinct tensile and compressive mechanical responses and strain over-limit characteristics of SRPE pipelines. The safety margin against code criteria and failure control mechanisms differ significantly under different fault angles. The findings can provide a theoretical basis for buckling safety evaluation and engineering protection of SRPE pipelines under diverse strike-slip fault conditions.

## 4. Conclusions

This study systematically investigates the mechanical behavior, failure mechanisms, and influencing factors of buried SRPE pipelines traversing strike-slip faults through finite element simulation. It conducts a comparative evaluation of the applicability of current design codes, leading to the following main conclusions:

1. Under strike-slip fault dislocation, the buckling failure of SRPE pipelines presents distinct stage characteristics. Pipeline deformation starts with elastic bending and local stress concentration under small displacement, develops into progressive plastic buckling under medium displacement, and eventually induces section distortion and global instability under large displacement. The failure process follows a sequential evolution mode: initial plastic deformation and sectional distortion of the PE matrix trigger subsequent yielding of the steel frame, ultimately leading to structural instability and pipeline leakage.
2. Fault intersection angle, steel frame diameter and backfill soil type are the key factors dominating the buckling resistance of SRPE pipelines. The fault angle determines the mechanical response mode, and the 150° transverse extrusion condition is verified as the most unfavorable load case. The optimal enhancement of buckling performance is achieved when the steel frame diameter ranges from 2.0 to 2.5mm. Among typical soil types, loess provides the best lateral confinement, while sandy soil induces the most severe buckling risk with weak restraint capacity. The dominant failure mechanism transforms significantly with fault angle variation. Small-angle dislocation at 30° leads to tension-dominated failure, and the critical tensile strain exceeds the thresholds of existing specifications. As the angle increases to 150°, the mechanical mode shifts from tension dominance to compression dominance, and compressive buckling becomes the primary failure mode. Comparative verification with CSA Z662, GB 50470 and EN 13476−3 indicates that the conventional constant strain criteria fail to characterize the angle-dependent mechanical effect, resulting in variable safety margins under different fault angles. It is essential to revise the current buckling evaluation standards according to practical fault angles for accurate engineering safety assessment.
3. This study focuses on the mechanical behavior of SRPE pipelines during the primary buckling stage, while the interfacial debonding behavior and post-buckling performance under large deformation are not incorporated. Severe fault displacement may induce interface separation between the steel frame and PE matrix, which alters the cooperative bearing mechanism and failure evolution path. Future research will concentrate on the interfacial damage evolution and post-buckling mechanical characteristics of composite pipelines, so as to improve the fault-induced failure evaluation system of SRPE pipelines and provide theoretical references for engineering disaster prevention and protection.The complete experimental procedure has been uploaded to protocols.io and is freely available:
